# COVID‐19 in a child with primary antibody deficiency

**DOI:** 10.1002/ccr3.3643

**Published:** 2020-12-08

**Authors:** Hamid Ahanchian, Nasrin Moazzen, Majid Sezavar, Negar Khalighi, Maryam Khoshkhui, Mohammad Hassan Aelami, Nasrin Sadat Motevalli Haghi, Nima Rezaei

**Affiliations:** ^1^ Allergy Research Center Mashhad University of Medical Sciences Mashhad Iran; ^2^ Clinical Research Development Unit of Akbar hospital Mashhad University of medical Sciences Mashhad Iran; ^3^ Department of Pediatrics, Faculty of Medicine Mashhad University of Medical Sciences Mashhad Iran; ^4^ Department of Pediatrics and Hand Hygiene and Infection Control Research Center Imam Reza Hospital, Mashhad University of Medical Sciences Mashhad Iran; ^5^ Research Center for Immunodeficiencies, Children's Medical Center Tehran University of Medical Sciences Tehran Iran; ^6^ Department of Immunology, School of Medicine Tehran University of Medical Sciences Tehran Iran; ^7^ Network of Immunity in Infection, Malignancy and Autoimmunity (NIIMA) Universal Scientific Education and Research Network (USERN) Tehran Iran

**Keywords:** COVID‐19, primary antibody deficiency, primary immunodeficiency, SARS‐Cov2

## Abstract

Although presentation of COVID‐19 in patients with immunodeficiency could be mild, it should not be missed, while early diagnosis and appropriate treatment can survive infected patients. Because even severe infections in PID patients may be presented with few symptoms and signs, this diagnosis should be considered in those immunocompromised patients who have exacerbating preexisting symptoms.

## Graphical abstract

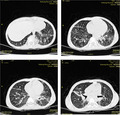

## 1 BACKGROUND

The novel coronavirus (COVID‐19) pandemic has made millions of people ill so far. Clinical manifestations and the natural history of infection have a very wide range. Although COVID‐19 has affected children less than adults, the presentation especially in those with underlying diseases should not be neglected. Herein, we report a case of primary antibody deficiency, who was infected with COVID‐19. The patient suffered from mild rhinorrhea and modestly increased in productive cough, but the real‐time reverse transcription polymerase chain reaction was positive for COVID‐19. His manifestation improved after a few days of antibiotic administration, and in the follow‐ups during 6 months, there were no symptoms of recurrence. Although presentation of COVID‐19 in patients with immunodeficiency could be mild, it should not be missed. Early diagnosis and appropriate treatment would help the infected patients to regain their health and survive the illness caused by COVID‐19. Even severe infections in the PID patients might present only a few symptoms and signs; therefore, the possibility of COVID‐19 infection should be considered in those immunocompromised patients whose preexisting symptoms have worsened.

New emerging coronavirus disease 2019 (COVID‐19) caused by the severe acute respiratory syndrome coronavirus 2 (SARS‐CoV‐2) has created a global concern,^1^ especially for those with underlying diseases including the immunocompromised individuals.

The primary immunodeficiency diseases (PIDs) are a heterogeneous group of disorders and an increased susceptibility to infections or predisposition to unusual infections is one of the main features in majority of the PID patients. In Iran, there are more than 3000 diagnosed patients with PIDs, one‐third of them suffer from the primary antibody deficiencies (PADs).^2^

Herein, we present an Iranian case of the primary antibody deficiency who was infected by SARS‐CoV‐2.

## 2 CASE PRESENTATION

An 8‐year‐old boy was diagnosed with PAD in our clinic. He had a history of recurrent otitis media and pneumonia since early infancy leading to multilobar bronchiectasis. The laboratory studies showed normal sweat test, normal IgG level; but low IgM and IgA levels, and low‐specific antibodies responses (Table 1), which could be compatible with a diagnosis of common variable immunodeficiency (CVID) associated with specific antibody deficiency. Intravenous immunoglobulin (IVIG) replacement therapy (400 mg/kg per month) was started for the patient, which significantly improved the clinical condition and reduced the episodes of respiratory tract infections during the last 18 months. Nebulized aminoglycoside and salbutamol were also administered at home.

**TABLE 1:** The results of laboratory test in the patient with primary antibody deficiency

WBC	10 350
Neutrophil	35.8%
Lymphocyte	33.6%
Eosinophil	22.8%
Monocyte	7.6%
Hemoglobin	10.2 (g/L)
Platelet‐	679 000
IgG	1622 (mg/dL)
IgA	2 (mg/dL)
IgM	38 (mg/dL)
Isohemagglutinins	Negative
Anti‐tetanus	<0.1 (IU/mL)
Anti‐diphtheria	0.1 (IU/mL)
Anti‐hepatitis B	2 (mlu/mL)
CD3 + T cells	67%
CD4 + T cells	20.3%
CD8 + T cells	47.1%
CD19 + B cells	18%
PPD test (mm)	Normal

**FIGURE 1:**
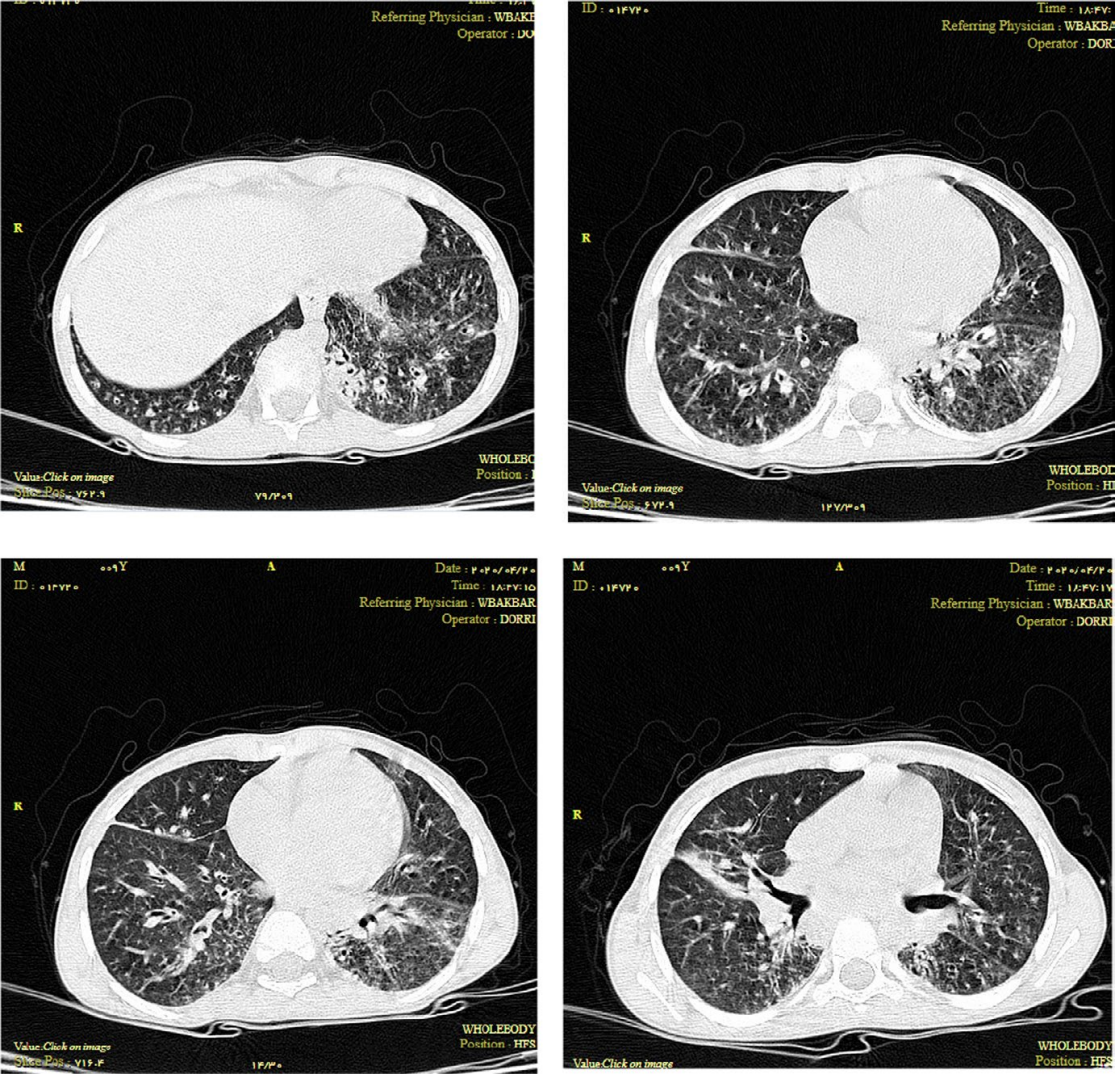
Computed tomography scan revealed evidence of bronchiectasis and collapse consolidations in an 8‐y‐old boy with primary antibody deficiency

**FIGURE 2:**
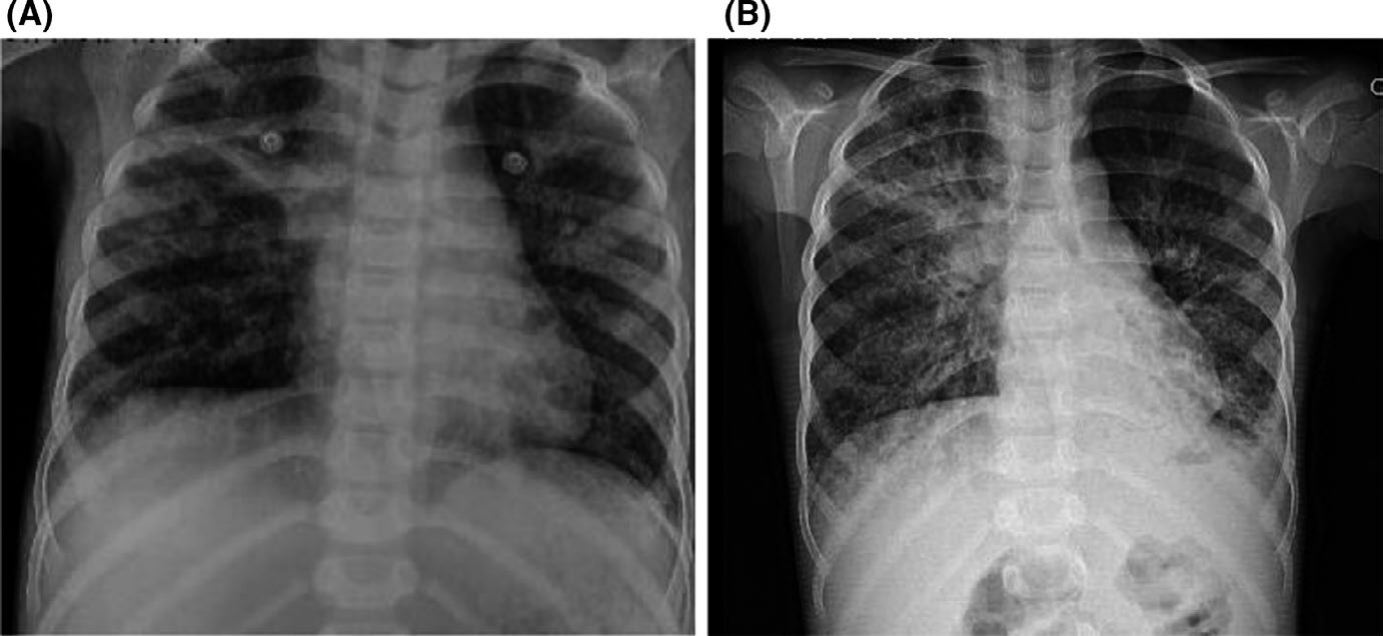
Chest X‐ray of 8‐y‐old boy with primary antibody deficiency. A, 1 y ago. B, recent admission

After 1 year of regular treatment, we visited the patient in our Outpatient Immunodeficiency Clinic (April 24, 2020), as the routine monthly follow‐up. He had mild rhinorrhea and modestly increased productive cough, starting a few days prior to his visit on April 24. He had no fever or exhibited any other symptoms. The mild rhinorrhea and modest productive cough symptoms were absent in his last IVIG infusion in late March of 2020. In the physical examination on late April, fine crackles were heard at the base of both lungs. He had tachypnea and mild suprasternal retraction, and the chest radiography showed perihilar consolidation with right middle lobe prominence (Figures 1, 2). We suspected an infection by the SARS‐CoV‐2, hence we requested a real‐time reverse transcription polymerase chain reaction (rRT‐PCR) for it. The result was positive.

Consequently, the patient was hospitalized to receive meropenem (100 mg/kg/day divided q 8 hours), clindamycin (40 mg/kg/day divided q 8 hours), and hydroxychloroquine (10 mg/kg/day stat and then 5 mg/kg/day for 5 days). His oxygen saturation was normal and he did not need supplemental oxygen. We administered it “as needed”. He was admitted to the COVID‐19 ward and there was no need to admit him to the ICU.

After 7 days of hospitalization, his condition was improved and the patient was discharged. After 6 months of follow‐ups, he is well without any complication. He receives IVIG every 4 weeks, and due to the absence of symptom exacerbation, we have not repeated rRt‐PCR for COVID‐19.

## 3 DISCUSSION

It has been globally acknowledged that SARS‐CoV‐2 infection has milder course and better prognosis in children compare to adults.^3^ But children with the immunodeficiency have a predisposing condition for COVID‐19. A multi‐center international report in September 2020, 94 patients with inborn error of immunity was done among them, 53 three patients (56%) had the primary antibody deficiency. More than 30% of all patients had common variable immune deficiency; but four deaths (45% of all deaths) were occurred in them. Although their median age was 45‐54 years, and most probably they had pre‐exiting diseases.^4^ Although immunocompromised patients may be more predisposed to the direct viral insult that could lead to organ damage and precede to morbidity and mortality, those with combined immunodeficiency are at a higher risk.^5^

On the other hand, most of the death due to the COVID‐19 is accompanied with the immune activation and cytokine storm, which seems to be impaired in the immunodeficient patients.^6^ As it is well known the immune system is affected by the COVID‐19,^7^ and a defective immune system could predispose individuals to viral infections, including COVID‐19. There is no approved treatment for the SARS‐CoV‐2 infection for children and usually the recommended treatments are merely supportive. In the recent report of COVID‐19 in patients with inborn error of immunity, more than half of the patients have received antibiotics: 33% hydroxychloroquine/chloroquine and about ten percent immunoglobulin.^4^

In the presented case concerning an 8‐year boy with an antibody deficiency, the SARS‐CoV‐2 infection presented itself with cough and rhinorrhea, which both are unusual in the SARS‐Cov‐2 infected children with normal immune system. This case emphasizes that healthcare workers should not ignore COVID‐19 infection in patients with PID, even if it is exhibited with the rare symptoms of COVID‐19. Our knowledge about the disease course and its possible complications is very limited. Hence, it is important to evaluate these infected PID patients for possible acute and chronic complications.

Considering that our patient is living with his parents and one older brother, and despite the fact that he has had appropriate hand hygiene and mask and had stayed at home for 2 months (when the COVID‐19 was at its peak in Iran); but because his older brother did not care much and came into contact with other people without any limitation, the young man got infected eventually. None of the family members have had any symptoms or positive rRt‐PCR test of SARS‐CoV‐2 infection. It is supposed that due to more susceptibility of infection in these population, all of family members are obligated to keep social distancing and maintain the personal hygiene standard.

## 4 CONCLUSION

Severe acute respiratory syndrome coronavirus 2 infection presentation in immunocompromised patients, including PIDs, may be mild like common cold or only exacerbating preexisting symptoms, but due to even severe infections in PID patients may presented with few symptoms and signs, this diagnosis should be considered in those immunocompromised patients who have exacerbating preexisting symptoms.

## CONFLICT OF INTERESTS

None.

## AUTHOR CONTRIBUTIONS

All the authors were involved in recruiting data for the paper. HA, NM, and NR drafted the report, MK, MHA, and NSMH critically revised the manuscript. All the authors approved the final draft.

## ETHICAL APPROVAL

This study was approved by the Ethics committee of Tehran University of Medical Sciences.

## CONSENT FOR PUBLICATION

All the authors approved submission of this paper to this journal.

## Data Availability

Data of patient is available, if needed.
